# Accuracy of color Doppler ultrasonography and magnetic resonance imaging in diagnosis of placenta accreta: A survey of 82 cases

**Published:** 2017-04-10

**Authors:** Sedigheh Ayati, Leila Pourali, Masoud Pezeshkirad, Farokh Seilanian Toosi, Sirous Nekooei, Mohammad Taghi Shakeri, Mansoureh Sadat Golmohammadi

**Affiliations:** 1 *Department of Obstetrics and Gynecology, School of Medicine, Mashhad University of Medical Sciences, Mashhad, Iran.*; 2 *Department of Radiology, School of Medicine, Mashhad University of Medical Sciences, Mashhad, Iran.*; 3 *Department of Biostatistics, School of Medicine, Mashhad University of Medical Sciences, Mashhad, Iran.*

**Keywords:** Placenta previa, Color Doppler ultrasonography, Magnetic resonance imaging

## Abstract

**Background:**

Placenta adhesive disorder (PAD) is one of the most common causes of postpartum hemorrhage and peripartum hysterectomy. The main risk factors are placenta previa and prior uterine surgery such as cesarean section. Diagnosis of placenta adhesive disorders can lead to a decrease of maternal mortality and morbidities.

**Objective:**

The purpose of this study was to compare the accuracy of color Doppler ultrasonography and magnetic resonance imaging (MRI) in the diagnosis of PADs.

**Materials and Methods:**

In this is cross-sectional study, Eighty-two pregnant women who were high risk for PAD underwent color Doppler ultrasound and MRI after 18 weeks of gestation. The sonographic and MRI findings were compared with the final pathologic or clinical findings. P<0.05 was considered statistically significant.

**Results:**

Mean maternal age was 31.42±4.2 years. The average gravidity was third pregnancy. 46% of patients had placenta previa. The history of the previous cesarean section was seen in 79 cases (96%). The diagnosis of placenta adhesive disorder was found in 17 cases (21%). Doppler sonography sensitivity was 87% and MRI sensitivity was 76% (p=0.37). Doppler sonography specificity was 63% and MRI specificity was 83% (p=0.01).

**Conclusion:**

Women with high-risk factors for PAD should undergo Doppler ultrasonography at first. When results on Doppler sonography are equivocal for PAD, MRI can be performed due to its high specificity.

## Introduction

PAD is a catastrophic and life-threatening obstetric condition that is an important cause of intrapartum and postpartum hemorrhage and peripartum hysterectomy. The prevalence of placenta accreta was 1 in 30,000 deliveries in the United States in 1950. Recently, the prevalence of PAD has varied in different studies from 1 in 500 to 1 in 2500 deliveries (1, 2). During the past few decades, the rate of cesarean delivery in the United States has increased from 5.8% (1970) to 32.9% (2009), increasing the risk for the various types of PAD (3-5). 

Placentation abnormalities pose one of the most important current challenges in the field of obstetrics and gynecology. *Placental invasion* or an abnormal attachment of the placenta to the uterine wall is classified on the basis of the depth of invasion into the category of placenta accreta, placenta increta, or placenta percreta. *Placenta accreta* occurs when the placenta is abnormally adherent to the underlying decidua. *Placenta increta* occurs when the placenta penetrates to the myometrium, and *placenta percreta* is defined by the invasion of the placenta into the uterine serosa or surrounding organs. In literature, placenta adhesive disorder (PAD) refers to any degree of placental invasion (3). 

The incidence of PAD has increased approximately 13-fold in recent years because of the increased rate of cesarean delivery (3-5). The incidence of PAD also grows parallel with advanced maternal age (for which there is an increased need for cesarean delivery) (1).

The risk factors for PAD are: placenta previa, prior uterine surgery such as cesarean section, myomectomy, Asherman syndrome, endometrial defects due to vigorous curettage, corneal resection, anterior placenta, smoking, hypertension, pregnancy induced via in vitro fertilization (IVF), subserosal and submucosal myoma, and female fetus (6-13). Hysteroscopic surgery, endometrial ablation, and uterine artery embolization may also result in localized decidual defects and result in abnormal placentation (14).

Average intraoperative blood loss in women with PAD is between 3-5 liters and is the most common cause of peripartum hysterectomy because of placental invasion and uncontrolled hemorrhage (9, 15-17). 90% of patients with placenta accreta require the blood transfusion, and 40% require more than 10 units of packed red blood cells. The rate of maternal mortality reported with placenta accreta was 7% in one study (1). Other disorders related to PAD include intravascular coagulopathy, adult respiratory distress syndrome, renal failure, cystectomy, ureteral injury, deep vein thrombosis, sepsis, significant maternal morbidity, and admission to the intensive care unit (9, 15-18).

Accurate prenatal diagnosis of PAD allows optimal management, ensuring availability of blood components along with a skilled surgical team, anesthetist, and interventional radiologist. Transabdominal sonography is the primary technique used to rule out PAD; however, technical difficulties exist with this approach. Bladder distention and contraction of the myometrium, maternal obesity, and posterior placentation can create false positive results (19). 

The diagnosis of placenta accreta is usually made based on clinical history, imaging findings, and histological features. When abdominal ultrasonography cannot definitively exclude PAD as a diagnosis, the next imaging technique used is Doppler ultrasonography. Magnetic resonance imaging (MRI) in high-risk patients is a gold standard in antenatal diagnosis, but is not as available as (and is also more expensive than) ultrasonography (20).

The aim of this study was to evaluate the diagnostic value of color Doppler ultrasonography and MRI in pregnant women at high risk for PAD.

## Materials and methods

This cross-sectional study was performed from December 2012 to December 2013. Eighty-two pregnant women after 18 weeks of gestation were referred for suspected PADs to the academic hospitals of Mashhad University of Medical Sciences, Masshad, Iran. 

Inclusion criteria were pregnant women who had at least two of the following risk factors: placenta previa, prior uterine surgery (such as a previous cesarean section, uterine curettage, or myomectomy), high maternal age (≥35 yr), and high parity (≥5). Exclusion criteria were MRI contraindications, cardiac pacemaker, metal objects in the body, and patient’s refusal for MRI evaluation. 

Demographic characteristics including age, parity, gravidity, and gestational age was obtained. Each of these women at high risk for PAD was evaluated by two modalities, including magnetic resonance imaging and color Doppler ultrasonography. Color Doppler ultrasonography was performed only by one experienced radiologist using a GE version 23 ultrasound device (GE Electric Medical Systems, Milwaukee, Wisconsin, USA) in combination with an abdominal convex probe of 3.5 MHz. 

The US diagnostic criteria of PAD include loss of the normal hypoechoic retroplacental zone, disruption of the hyperechoic uterine serosa-bladder interface, intraplacental lacunae, and hypervascularity at the interface between the uterine serosa and the urinary bladder wall. MR images were evaluated by a radiologist experienced in placental MRI evaluation, and all images were read without knowledge of the results of the ultrasonography scans. No contrast medium was used. 

The MRI criteria for diagnosing PAD were: uterine bulge, disruption of the hyperechoic uterine serosa-bladder interface, intraplacental lacunae, and hypervascularity at the interface between the uterine serosa and the urinary bladder wall.

All women who were believed to have PAD were scheduled for elective cesarean delivery. Controlled conditions (such as blood components, skilled anesthetist, the surgical team [obstetrician/ gynecologist, surgeon, gynecologic oncologist, urologist, vascular surgeon] were planned for in advance and provided to prevent intraoperative blood loss.

After the surgery, PAD was diagnosed with difficulty in removing the placenta, uncontrolled bleeding after manual removal of the placenta, or on the basis of pathologic findings. 

**Ethical consideration**

The study protocol was approved by the ethics committee of Mashhad University of Medical Sciences (code number, IR.MUMS.REC.1393.134), and informed consent was obtained from all participants.

**Statistical analysis**

To compare between the groups, analysis of variance (ANOVA) (for normal data) or Kruskal-Wallis (for non-normal data) tests were used. The K2 test was used for qualitative variables. P<0.05 was considered statistically significant.

## Results

A total of 82 women at high risk of PAD were enrolled in this study. The mean maternal age was 31.42±4.2 yr (age range for the entire population, 20-40 yr). Among 82 women, 79 cases (96%) had a previous cesarean section; 30 (36.6%) one, 38 (46.3%) two, and 11 (13.4%) had three previous cesarean section. There were 24 women (29.3%) with the previous curettage; 18 (22%) had one, 4 (4.9%) two, and 2 (2.4%) had three previous curettages. Forty-six percent of patients had placenta previa according to Doppler sonography and MRI. 

For placental implantation, the anchoring villi attachment was at the anterior wall of the uterus in 46 cases, posterior in 15, fundal in 8, lateral in 6, anterolateral in 4, and dorsolateral in 2 cases. Color Doppler sonography showed positive findings for PAD in 39 cases (48%) and negative in 43 (52%) patients. On MRI, findings were positive in 24 cases (29%) and negative in 58 cases (71%). Doppler sonography sensitivity was 87% and MRI, 76% (p=0.37). Doppler sonography specificity was 63% and MRI, 83% (p=0.01). Overall, two techniques were not significantly different in terms of sensitivity; the specificity of MRI in recognition of PAD was significantly higher than that with Doppler sonography. 

Elective cesarean delivery was performed for all patients, and 17 women (21%) were identified as PAD. The management of 17 cases of PAD was performed by cesarean hysterectomy in 10 cases (59%), packing with gauze or uterine, and/or ovarian and /or hypogastric artery ligation in 7 patients (41%). In patients with PAD, 15 cases had placenta previa (88%); 7 (41.1%) had one, 8 (47%) patients had two and 2 (11.7%) had three previous cesarean section. No maternal mortality or morbidity occurred in these women.

**Table I.** Sensitivity and specifity of color Doppler sonography and MRI

**Variable **	**Color Doppler sonography**	**MRI**	**p-value**
Sencitivity	87	76	0.37
Specificity	63	83	0.01

Data presented as %.

MRI: magnetic resonance imaging

## Discussion

Our study showed that Doppler sonography and MRI without use of gadolinium appear to have similar sensitivity for diagnosis of placenta accreta, but the specificity of MRI was significantly higher than Doppler sonography..

Some studies comparing both imaging techniques have not demonstrated significant differences in sensitivity (11, 19, 21, 22). Warshak et al reported on 39 cases of placenta accreta (PA) confirmed by pathological examination for which ultrasound had a sensitivity of 77% and a specificity of 96%, whereas MRI with contrast had a sensitivity of 88% and a specificity of 100% (19). The difference in the specificity of MRI between studies could be due to the use of gadolinium, which enabled the boundary between the placenta and myometrium to be shown properly. The use of gadolinium in pregnancyis contraindicated and it just recommended by the European Medicine Agency only if the benefits outweigh the risks (23).

Using of gadolinium in pregnancy is controversial given that the molecule crosses the placenta, enters the fetal circulation, and is excreted by the fetal kidney (22). Other studies have reported that gadolinium causes nephrogenic fibrosis, renal failure, and even mental retardation in high doses (20). Performing MRI for PAD cases diagnosed by sonography could be another reason for the seemingly enhanced specificity of MRI. Elhawari *et al* reported that the sensitivity of ultrasonography was 82%, while MRI in the same cases demonstrated a sensitivity of 88%; their specificities were 89% and 86%, respectively (19). 

In their study, the differences in sensitivity and specificity between sonography and MRI did not meet statistical significance; both were higher than those presented in this study, potentially due to their inclusion criteria (presence of placenta previa in all cases). Placenta previa is recognized as one of the main risk factors for PAD, thereby increasing specificity. This may represent an unavoidable source of bias. The differences in the specificity of sonography could also be due to the small number of patients in their study. 

Two recently published systematic reviews have mentioned the accuracy of Doppler sonography and MRI for diagnosis of PAD (24, 25). D'Antonio et alreported a sensitivity of 90.7% for ultrasound and 94.4% for MRI, and a specificity of 96.9% and 84% for ultrasound and MRI, respectively (24). Meng et al reported that ultrasound had a sensitivity of 82% and specificity of 89%, while MRI had a sensitivity of 82% and a specificity of 88% (25). Both of these reviews included several studies and a large number of patients, and came to the same conclusion, namely that both imaging techniques had equal accuracy in diagnosis of invasive placentation. Although the percentage of correct diagnoses was enhanced in each of these studies, no differences existed in sensitivity or specificity between ultrasonography and MRI; the specificity of ultrasound and MRI are not the same in the current study. This difference may be due to the fact that they are systematic reviews; they did not consider studies clinically and methodologically, and ultrasonography and MRI were not applied to identical populations. 

Maher et al published a study in which specificity and sensitivity increased for ultrasonography as compared to MRI, potentially due to the use of transvaginal sonography (26). It has been suggested that transvaginal sonography may improve accuracy for antenatal diagnosis of placenta accreta by improving the near-field resolution (22). In the study of Maher et al, MRI was performed in patients for whom the ultrasound depiction was unclear; therefore, MRI added to the specificity of the diagnosis (26). 

Another study published by Lim et al reported that Doppler sonography had a sensitivity of 67% and specificity of 50%; while MRI had a higher sensitivity and specificity of 75% and 78%, respectively (27). compared with current study, *Lim et al* showed decreased sensitivity and specificity, possibly due to the fact that abdominal sonography was performed in the patient population. Furthermore, Lim obtained imaging at a different time (at 30 wk gestation or sooner) than in our study. They reported that placental hypointense bands in MRI increase specificity (27).

In another study, by Riteau et al, sonography and MRI were each performed in all patients, as with our study (28). Doppler sonography had a sensitivity of 100% and a specificity of 37.5%; MRI’s sensitivity was 76.9%, its specificity 50%. In comparison with our study, the sensitivity increased but the specificity decreased, potentially due to the standards by which sonography was presumed to show PAD were different from those in our study. The skill and experience of the sonographer in our study were different than those in Riteau et al (28).

The strengths of our study include the following: 1) its prospective design; 2) color Doppler ultrasonography was performed only by one experienced radiology, and MRI performed by another who was a professional in the field; each were blind to the results yielded by the other techniques; 3) the multidisciplinary approach of our study led to no mortality and minimal morbidity in patients with PAD; and 4) optimal sample size helps to the reliability of the study findings.

## Conclusion

Patients at high risk for PAD should undergo Doppler ultrasonography. When results on Doppler sonography are equivocal for PAD, MRI can be performed due to its high specificity. For the future, it is recommended to evaluate diagnosis of PAD by using transvaginal ultrasonography, and its accuracy for transabdominal color Doppler ultrasonography. 

## Note

***This article extracted from residential thesis. (Leila Pourali)***
